# Cabozantinib-Exposed Renal Cell Carcinoma Organoids Suggest Transcriptomic Associations with Treatment Resistance in Clear Cell and Nonclear Cell Tumors

**DOI:** 10.15586/jkc.v12i2.386

**Published:** 2025-05-05

**Authors:** Wesley H. Chou, Nicholas H. Chakiryan, George V. Thomas

**Affiliations:** 1 Department of Urology, Oregon Health & Science University, Portland, Oregon;; 2 Knight Cancer Institute, Oregon Health & Science University, Portland, Oregon;; 3 Department of Urology, Portland VA Medical Center, Portland, Oregon;; 4 Department of Pathology and Laboratory Medicine, Oregon Health & Science University, Portland, Oregon

**Keywords:** renal cell carcinoma, cabozantinib, tyrosine kinase inhibitor, organoid, single-cell RNA sequencing

## Abstract

While vascular endothelial growth factor tyrosine kinase inhibitors (VEGF-TKIs) are a mainstay of treatment for advanced renal cell carcinoma (RCC), mechanisms of resistance to VEGF-TKIs remain under ongoing investigation. To assess transcriptomic changes in clear-cell RCC (ccRCC) and non-ccRCC exposed to a VEGF-TKI, we analyzed differential single-cell gene expression in RCC tumor-organoids exposed to cabozantinib versus control solvent. In ccRCC organoid cells, *LRRC75A* was notably highly associated with cabozantinib exposure (log2 fold-change 2.18, detected proportion 0.52 vs. 0.23, false-detection rate adjusted p<0.001). Importantly, our findings were independently validated in a recent study of advanced ccRCC patients treated with cabozantinib, which demonstrated that higher *LRRC75A* expression was significantly associated with decreased tumor response and less robust reduction of VEGF expression. *LRRC75A* has been shown to mediate VEGF secretion in a separate study and may potentiate compensatory angiogenesis after cabozantinib exposure. Gene expression scores were then developed based on transcriptomic changes associated with cabozantinib exposure and applied to stage IV patients in several independent cohorts. Higher scores were significant predictors of worse overall survival in TCGA non-RCC patients and worse progression-free survival in JAVELIN Renal 101 ccRCC patients. Overall, this experiment represents an incremental step in a larger effort to elucidate resistance mechanisms to VEGF-TKIs.

## Introduction

Vascular endothelial growth factor tyrosine kinase inhibitors (VEGF-TKIs) is the backbone of therapy for patients with advanced renal cell carcinoma (RCC). Current indications for these agents include first-line therapy in combination with immune checkpoint inhibitors (ICIs) for advanced RCC, as second-line monotherapy for patients with clear-cell RCC (ccRCC) who progress on ICI, and as first-line monotherapy in patients who cannot receive ICIs (1).

Various mechanisms have been proposed for potential resistance to contemporary VEGF-TKIs in RCC, including bypassing of anti-angiogenesis via promotion of IL-6 and IL-8 secretion (2,3), upregulation of the c-Met receptor IRAK1 and downregulation of the tumor suppressor MCPIP1 (4), lysosomal sequestration and use of efflux transporters (5), among others. However, there have been comparatively few studies on TKI resistance mechanisms versus ICIs (6,7), and even fewer looking at specifically the TKI cabozantinib. Park and colleagues described a ccRCC and peripheral blood mononuclear cell co-culture experiment which identified helper T-cell subtypes that may mediate resistance to cabozantinib via an increase in pro-angiogenic factors (8). Outside of this report, there is an overall paucity of data describing potential mechanisms of resistance to cabozantinib in ccRCC and essentially none for non-ccRCC.

To further explore this topic, we sought to assess the transcriptomic changes that ccRCC and non-ccRCC tumor cells undergo when exposed to cabozantinib. Herein, we report the results of an experiment using RCC tumor-organoid models to determine differential single-cell gene expression between organoids treated with the TKI cabozantinib versus control dimethyl sulfoxide (DMSO) delivery solvent.

## Methods

Four pairs of RCC-derived organoids were treated with either DMSO (control) or cabozantinib for 48 hours. Two of these pairs were derived from ccRCC tumors (OHSU30, NCI350), whereas the other two pairs were non-ccRCC derived (OHSU29, NCI274). The OHSU29 organoid is a chromophobe RCC with sarcomatoid differentiation, whereas NCI274 is histologically RCC not otherwise specified with papillary features. Fresh, frozen organoid tissue was dissociated into single-cell suspensions which were then utilized for single-cell RNA sequencing with 10× chromium, a droplet-based gel bead barcoding platform, per manufacturer recommended protocols. Standard protocols for preprocessing, alignment to the hg19 reference genome, quality control, cell filtering, data normalization, and downstream analysis were completed using CellRanger (4), Seurat (5), and DEseq2 software packages (6), among others.

The ccRCC and non-ccRCC samples were split and independently integrated to control for batch effects. Transcriptomic data were transformed prior to dimensionality reduction and differentially expressed gene (DEG) analysis between treatment groups. Uniform manifold approximation and projection (UMAP) was used to visualize the data, split by ccRCC versus non-ccRCC organoid status. Results were also stratified by cabozantinib versus DMSO exposure. DEG results were filtered by absolute log2 fold change (FC) levels >1.0 and false discovery rate (FDR)-adjusted p-values <0.05 to select the reported transcriptomic associations. The Kyoto Encyclopedia of Genes and Genomes (KEGG) 2021 human gene pathway database was utilized for biologic pathway analysis using the Enrichr package (9,10).

Given the significantly increased expression of *LRRC75A* in the ccRCC organoids exposed to cabozantinib, we performed external independent validation based on RNA-seq data from Bilen and colleagues (11). In this phase 2 trial, 17 patients with locally advanced ccRCC were treated with cabozantinib prior to surgical resection. Normalized count expression levels of *LRRC75A* after cabozantinib exposure were correlated with tumor shrinkage. Post-cabozantinib *LRRC75A* expression was correlated with objective response rates as defined by RECIST criteria, as well as based on rhabdoid versus sarcomatoid histology. Changes in plasma levels of various angiogenesis-associated molecules pre- and post-cabozantinib exposure were additionally correlated with post-cabozantinib *LRRC75A* expression levels. Spearman coefficients and Wilcoxon p-values were used to assess these relationships.

Using the DEG results, two differential gene expression scores were developed based on exposure to cabozantinib, one for ccRCC and another for non-ccRCC. The score was created by subtracting the geometric mean of negatively associated genes from the geometric mean of positively associated genes, one for ccRCC and another for non-ccRCC-associated genes. Higher scores are indicative of transcriptomic changes associated with cabozantinib exposure in the organoid experiment.

These scores were then applied to RNA-seq data from several independent cohorts. The ccRCC-derived score was applied to patients with stage IV ccRCC from The Cancer Genome Atlas Kidney Renal Cell Clear Carcinoma (TCGA KIRC) (12) as well as patients with stage IV ccRCC from the JAVELIN Renal 101 phase III randomized controlled trial (13). For the JAVELIN Renal 101 patients, analysis was stratified between the TKI-only (sunitinib) and TKI and ICI (axitinib and avelumab) arms. The non-ccRCC-derived score was applied to patients with stage IV non-ccRCC from TCGA Kidney Renal Papillary Cell Carcinoma (KIRP) (14).

TCGA data were limited to specimens from treatment-naïve patients with year of diagnosis from 2005 to 2010. Given that TKIs were the main therapy for advanced RCC during that time period, we felt that treatment-naïve patients would have been highly likely to have received TKI therapy. Thus, the gene score derived from cabozantinib-exposed ccRCC organoids was felt to be applicable to this population (13). Kaplan–Meier survival distributions with log-rank tests were performed based on scoring for overall survival in the TCGA cohorts and progression-free survival in the JAVELIN Renal 101 arms.

## Results

**Figure 1:**
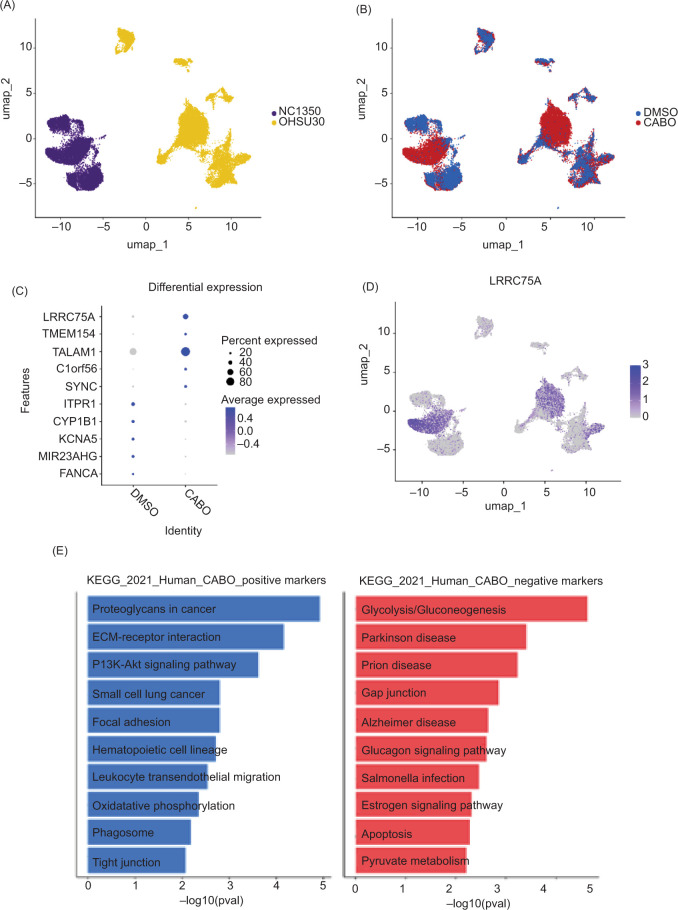
(A) UMAP projection of single cells from the ccRCC organoids NCI350 and OHSU30. (B) UMAP projection of single cells from ccRCC organoids stratified by the treatment group. (C) Top differentially expressed genes in ccRCC organoids stratified by the treatment group. (D) UMAP projection of single cells from ccRCC organoids with normalized expression of *LRRC75A* demonstrating greater expression with cabozantinib exposure. (E) KEGG pathway analysis of ccRCC organoids based on cabozantinib exposure.

After completion of quality control measures, 34,513 ccRCC organoid cells were included for analysis (Figure 1A and 1B). Among ccRCC samples, several genes demonstrated statistically significant differential expression between treatment groups (*LRRC75A, TMEM154, TALAM1, C1orf56, SYNC, ITPR1, CYP1B1, KCNA5, MIR23AHG*, and *FANCA*; Figure 1C). Most notably, *LRRC75A* was both visually and statistically highly associated with cabozantinib exposure to a substantially greater extent than the other identified genes (log2 FC 2.18, detected proportion 0.52 vs. 0.23, FDR-adjusted p<0.001; Figure 1D). KEGG pathway analysis identified “proteoglycans in cancer” and “ECM receptor interaction” as the two pathways most strongly associated with cabozantinib exposure among the ccRCC organoids (Figure 1E**)**.

**Figure 2:**
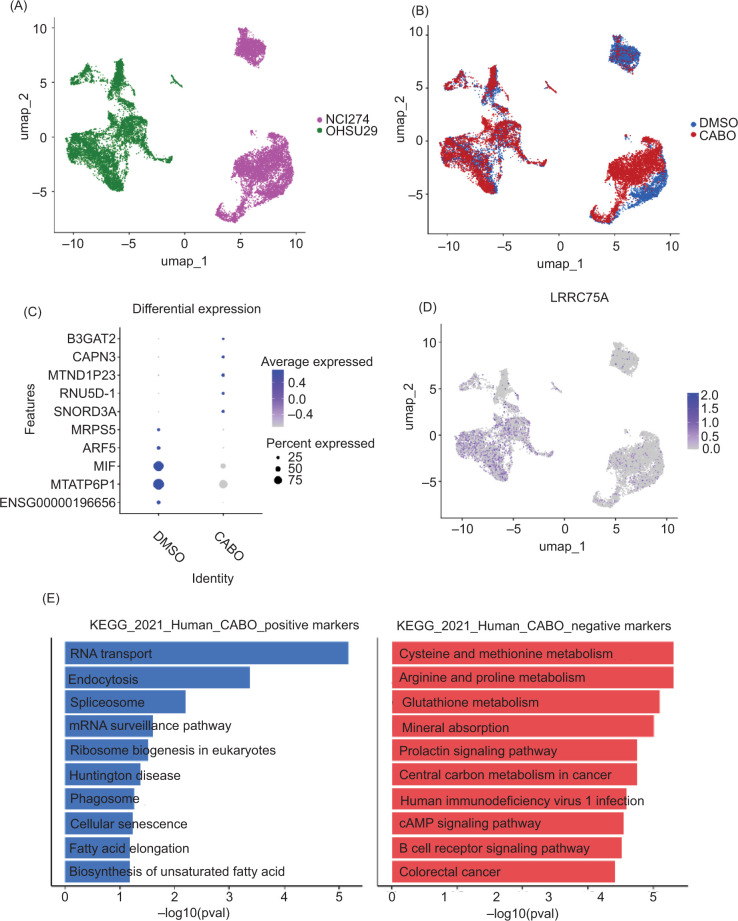
(A) UMAP projection of single cells from the non-ccRCC organoids NCI274 and OHSU 29. (B) UMAP projection of single cells from non-ccRCC organoids stratified by the treatment group. (C) Top differentially expressed genes in non-ccRCC organoids stratified by the treatment group. (D) UMAP projection of single cells from non-ccRCC organoids with normalized expression of *LRRC75A*. (E) KEGG pathway analysis of non-ccRCC organoids based on cabozantinib exposure.

After completion of quality control measures, 19,113 non-ccRCC organoid cells were included for analysis (Figure 2A and 2B). Among non-ccRCC samples, several genes demonstrated statistically significant differential expression between treatment groups (*B3GAT2, CAPN3, MTND1P23, RNU5D-1, SNORD3A, MRPS5, ARF5, MIF, MTATP6P1*, and *ENSG00000196656*; Figure 2C). None of these genes exhibited uniquely substantial treatment association over the other genes in this set. No significant change in *LRRC75A* expression was seen in the non-ccRCC organoid samples (log2 FC 0.01, detected proportion 0.181 vs. 0.116, FDR-adj. p=0.8; Figure 2D). KEGG pathway analysis identified “RNA transport” and “endocytosis” as the two pathways mostly strongly associated with cabozantinib-exposure among the non-ccRCC organoids (Figure 2E).

**Figure 3:**
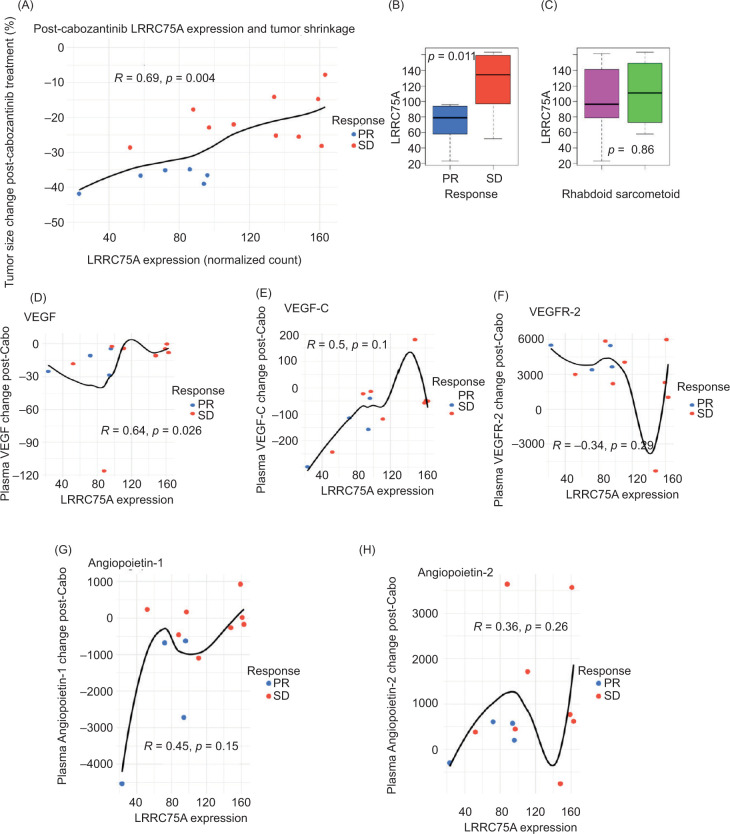
LRRC75A independent external validation using data from Bilen et al. (2025). (A) Scatter plot comparing post-cabozantinib LRRC75A RNAseq normalized count expression levels with tumor shrinkage percentage after 12 weeks of cabozantinib treatment, among 16 patients with complete data. Spearman correlation and p-value reported in plot. (B) Box plot comparing post-cabozantinib LRRC75A expression by RECIST criteria. Wilcoxon p-value reported in plot. (C) Box plot comparing post-cabozantinib LRRC75A expression by rhabdoid/sarcomatoid histologic status. Wilcoxon p-value reported in plot. (D–F) Scatter plots comparing post-cabozantinib LRRC75A expression levels with changes in pre- vs. post-cabozantinib treatment plasma levels of angiogenesis-related molecules, among 12 patients with complete data. Spearman correlation and p-value reported in plot.

Independent validation of *LRRC75A* expression in patients with advanced ccRCC treated with cabozantinib prior to surgical resection was performed. Higher levels of post-cabozantinib *LRRC75A* expression were significantly associated with decreased relative shrinkage in tumor size (Figure 3A, p=0.004), while patients with partial response had significantly lower *LRRC75A* expression compared to those with stable disease (Figure 3B, p=0.011); there was not significant variation in *LRRC75A* expression based on rhabdoid versus sarcomatoid histology (Figure 3C). Relative changes in several molecules associated with angiogenesis were evaluated in relation to post-cabozantinib *LRCC75A* expression levels (Figure 3D–3H). Only VEGF was noted to have a significant relationship, with smaller decreases in VEGF noted in patients with higher *LRRC75A* expression levels (p=0.026).

**Figure 4:**
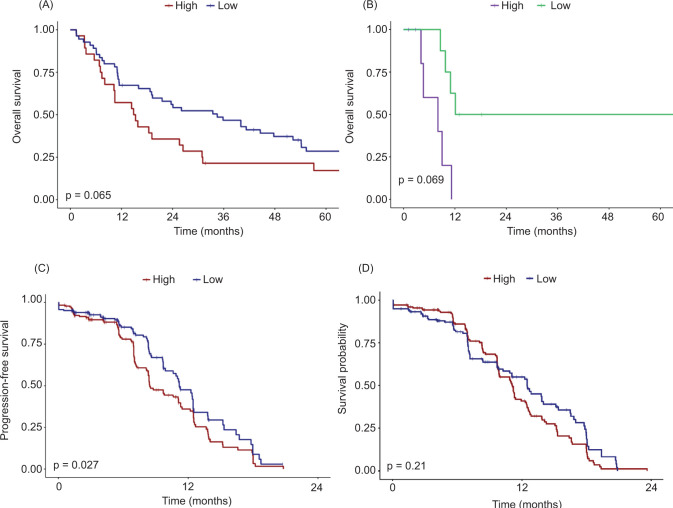
Kaplan–Meier distributions based on cabozantinib differential gene expression scores from ccRCC organoids for (A) overall survival in stage IV ccRCC patients from The Cancer Genome Atlas Kidney Renal Clear Cell Carcinoma (TCGA KIRC) and (B) overall survival in stage IV non-ccRCC patients from TCGA Kidney Renal Papillary Cell Carcinoma (TCGA KIRP). Gene expression scores based on non-ccRCC organoids were then used to create Kaplan–Meier distributions for progression-free survival in stage IV ccRCC patients from the JAVELIN Renal 101 phase 3 randomized controlled trial treated with (C) sunitinib only, as well as (D) axitinib and avelumab.

After generation of differential gene expression scores based on cabozantinib exposure from the organoid experiment, Kaplan–Meier survival distributions showed associations between higher scores and worse clinical outcomes. While this did not reach significance in the TCGA KIRC cohort (Figure 4A, p=0.065), it was demonstrated in the TCGA KIRP (Figure 4B, p=0.0069) and JAVELIN Renal 101 TKI-only (Figure 4C, p=0.027) cohorts. No significant association was seen between this gene score and the JAVELIN Renal 101 TKI and ICI arm (Figure 4D, p=0.21).

## Discussion

We sought to analyze potential mechanisms of VEGF-TKI resistance in RCC, specifically toward cabozantinib. In this scRNA-seq analysis, we identified transcriptomic associations with cabozantinib exposure in ccRCC and non-ccRCC tumor organoids. Gene scores based on these associations demonstrated translation to worsened survival outcomes among several independent cohorts, further suggesting association with treatment resistance.

*LRRC75A* emerged as a particularly notable signal, with the cabozantinib-exposed ccRCC organoids exhibiting significantly increased expression. We also found *LRRC75A* has not been previously described in the context of RCC. A separate study of mesenchymal stem cells found that under ischemic conditions, LRRC75A expression mediated VEGF secretion (15). Thus, *LRRC75A* may potentiate compensatory angiogenesis in cabozantinib-exposed ccRCC and VEGF-TKI resistance more generally. This finding was not reproduced in the non-ccRCC organoids, possibly reflecting their decreased propensity to exhibit molecular alterations in angiogenesis and hypoxia-ischemia pathways than ccRCC tumors. Separate external validation in a ccRCC patient cohort treated with cabozantinib found that higher *LRRC75A* expression was significantly associated with poorer tumor response and smaller decreases in VEGF expression, lending credence to our findings.

We also reviewed if the other gene signals from our analysis could have some plausible associations with resistance mechanisms to cabozantinib. Regarding upregulated genes in the ccRCC organoid subset, several have been implicated in the promotion of other nonrenal malignancies. Specifically, *TALAM1* has been shown to facilitate motility of breast cancer cells within in vitro as well as in vivo migration assays utilizing immunocompromised mice (16). *C1orf56* has also been implicated in the development of lymphomas in murine models and is also overexpressed in many human lymphomas (17). Finally, *SYNC* is elevated in human gastric cancers and interestingly has also been correlated to poorer survival outcomes and infiltration of M2-polarized tumor-associated macrophages (18). On the other hand, *TMEM154* has not been previously associated with tumorigenesis or cancer treatment resistance. In reviewing the upregulated genes for the non-ccRCC organoid subset, we did not find any existing research associating their overexpression with malignancy.

With regard to downregulated genes within the subset of ccRCC organoids, *ITPR1* has been shown to protect RCC cell lines against natural killer cell–mediated death (19). Supposing that *ITPR1* is truly protective of RCC cells, its downregulation in ccRCC organoids after cabozantinib treatment could be indicative of reliance on separate pathways for survival or suggestive that it is not critical for tumor survival. Separately, suppression of *KCNA5* in Ewing sarcoma and neuroblastoma cell lines has been shown to contribute to the survival of these malignant cells under hypoxic conditions (20), which would make more intuitive sense in the context of ccRCC organoid survival.

Interestingly, for downregulated genes in the non-ccRCC organoid subset, both low expression of *MRPS5* and *MIF* have been associated with poorer oncologic outcomes in patients with ccRCC (21, 22). Given that these studies only focused on patients with ccRCC, it is not clear if the downregulation of these genes may play a role in the aggressiveness of non-ccRCC as well.

To further explore if these DEGs translated to clinical outcomes, we used the transcriptomic associations to generate gene scores based on organoid exposure to cabozantinib and applied them to several independent cohorts of patients with stage IV RCC. Higher scores, indicative of cabozantinib-exposure in the organoid experiment, were associated with worse survival outcomes in both ccRCC and non-ccRCC patients. This stratification appeared particularly robust in the TCGA KIRP group, although this is a very small cohort. Interestingly, within the JAVELIN Renal 101 group, the gene score only significantly stratified progression-free survival in the TKI-only arm, suggesting that the addition of ICI may have negated some of the resistance mechanisms to TKIs.

## Conclusion

This experiment overall represents an incremental step in a larger effort to elucidate resistance mechanisms to VEGF-TKIs. Given that our current experiment was not designed to assess downstream molecular effects of the DEGs, further experimentation is required to determine the validity and implication of our findings.

## References

[1] Renal Cell Carcinoma. Uroweb [Internet]. [cited 2024 Nov 1]. Available from: https://uroweb.org/guidelines/renal-cell-carcinoma/chapter/disease-management

[2] Ishibashi K, Koguchi T, Matsuoka K, Onagi A, Tanji R, Takinami-Honda R, et al. Interleukin-6 induces drug resistance in renal cell carcinoma. Fukushima J. Med. Sci. 2018;64(3):103–10. 10.5387/fms.2018-15 30369518 PMC6305783

[3] Huang D, Ding Y, Zhou M, Rini BI, Petillo D, Qian CN, et al. Interleukin-8 mediates resistance to antiangiogenic agent sunitinib in renal cell carcinoma. Cancer Res. 2010 Feb 1;70:1063–71. 10.1158/0008-5472.CAN-09-3965 20103651 PMC3719378

[4] Marona P, Górka J, Kwapisz O, Jura J, Rys J, Hoffman RM, et al. Resistance to tyrosine kinase inhibitors promotes renal cancer progression through MCPIP1 tumor-suppressor downregulation and c-Met activation. Cell Death Dis. 2022 Sep 22;13(9):1–17. 10.1038/s41419-022-05251-4 PMC9500022 36138026

[5] Giuliano S, Cormerais Y, Dufies M, Grépin R, Colosetti P, Belaid A, et al. Resistance to sunitinib in renal clear cell carcinoma results from sequestration in lysosomes and inhibition of the autophagic flux. Autophagy. 2015 Aug 27;11(10):1891–904. 10.1080/15548627.2015.1085742 26312386 PMC4824581

[6] Lindner AK, Pichler M, Thurnher M, Pichler R. Targeting c-Met to improve immune checkpoint inhibition in metastatic renal cell carcinoma. Eur. Urol. 2022 Jan 1;81(1):1–2. 10.1016/j.eururo.2021.10.025 34794854

[7] Derosa L, Routy B, Fidelle M, Iebba V, Alla L, Pasolli E, et al. Gut bacteria composition drives primary resistance to cancer immunotherapy in renal cell carcinoma patients. Eur. Urol. 2020 Aug 1;78(2):195–206. 10.1016/j.eururo.2020.04.044 32376136

[8] Park KY, Hefti HO, Liu P, Lugo-Cintrón KM, Kerr SC, Beebe DJ. Immune cell mediated cabozantinib resistance for patients with renal cell carcinoma. Integr. Biol. 2021 Dec 21;13(11):259–68. 10.1093/intbio/zyab018 PMC8730366 34931665

[9] Kanehisa M, Sato Y, Kawashima M, Furumichi M, Tanabe M. KEGG as a reference resource for gene and protein annotation. Nucleic Acids Res. 2016 Jan 4;44(D1):D457–62. 10.1093/nar/gkv1070 26476454 PMC4702792

[10] Kuleshov MV, Jones MR, Rouillard AD, Fernandez NF, Duan Q, Wang Z, et al. Enrichr: a comprehensive gene set enrichment analysis web server 2016 update. Nucleic Acids Res. 2016 Jul 8;44(W1):W90–97. 10.1093/nar/gkw377 27141961 PMC4987924

[11] Bilen MA, Vo BT, Liu Y, Greenwald R, Davarpanah AH, McGuire D, et al. Neoadjuvant cabozantinib for locally advanced nonmetastatic clear cell renal cell carcinoma: a phase 2 trial. Nat. Cancer. 2025 Mar 6(3):432–44. 10.1038/s43018-025-00922-5 40016487

[12] TCGA-KIRC [Internet]. The Cancer Imaging Archive (TCIA). [cited 2025 Feb 11]. Available from: https://www.cancerimagingarchive.net/collection/tcga-kirc/

[13] Motzer RJ, Penkov K, Haanen J, Rini B, Albiges L, Campbell MT, et al. Avelumab plus axitinib versus sunitinib for advanced renal-cell carcinoma. N. Engl. J. Med. 2019 Mar 21;380(12):1103–15. 10.1056/NEJMoa1816047 30779531 PMC6716603

[14] TCGA-KIRP [Internet]. The Cancer Imaging Archive (TCIA). [cited 2025 Feb 11]. Available from: https://www.cancerimagingarchive.net/collection/tcga-kirp/

[15] Miura T, Kouno T, Takano M, Kuroda T, Yamamoto Y, Kusakawa S, et al. Single-cell RNA-seq reveals LRRC75A-expressing cell population involved in VEGF secretion of multipotent mesenchymal stromal/stem cells under ischemia. Stem Cells Transl. Med. 2023 Jun 15;12(6):379–90. 10.1093/stcltm/szad029 37263619 PMC10267575

[16] Gomes CP, Nóbrega-Pereira S, Domingues-Silva B, Rebelo K, Alves-Vale C, Marinho SP, et al. An antisense transcript mediates MALAT1 response in human breast cancer. BMC Cancer. 2019 Aug 5;19:771. 10.1186/s12885-019-5962-0 31382922 PMC6683341

[17] Hlady RA, Novakova S, Opavska J, Klinkebiel D, Peters SL, Bies J, et al. Loss of Dnmt3b function upregulates the tumor modifier Ment and accelerates mouse lymphomagenesis. J. Clin. Invest. 2012 Jan 3;122(1):163–77. 10.1172/JCI57292 22133874 PMC3248285

[18] Wang D, Deng L, Xu X, Ji Y, Jiao Z. Elevated SYNC expression is associated with gastric tumorigenesis and infiltration of M2-polarized macrophages in the gastric tumor immune microenvironment. Genet. Test Mol. Biomark. 2021 Mar 25(3):236–46. 10.1089/gtmb.2020.0131 33734892

[19] Messai Y, Noman MZ, Hasmim M, Janji B, Tittarelli A, Boutet M, et al. ITPR1 protects renal cancer cells against natural killer cells by inducing autophagy. Cancer Res. 2014 Nov 30;74(23):6820–32. 10.1158/0008-5472.CAN-14-0303 25297632

[20] Ryland KE, Svoboda LK, Vesely ED, McIntyre JC, Zhang L, Martens JR, et al. Polycomb-dependent repression of the potassium channel-encoding gene KCNA5 promotes cancer cell survival under conditions of stress. Oncogene. 2015 Aug 27;34(35):4591–600. 10.1038/onc.2014.384 25435365 PMC4451446

[21] Yang X, Han B, Xie Q, Li Y, Li Q, Hu X, et al. Low expression of mitochondrial ribosomal protein S5 is associated with poor prognosis in patients with clear cell renal cell carcinoma. Appl. Immunohistochem. Mol. Morphol. AIMM. 2025 Jan 1;33(1):22–8. 10.1097/PAI.0000000000001236 39636315 PMC11614452

[22] An HJ, Koh HM, Lee JS, Song DH. Prognostic role of macrophage migration inhibitory factor in patients with clear cell renal cell carcinoma. Medicine (Baltimore). 2020 Dec 11;99(50):e23277. 10.1097/MD.0000000000023277 33327252 PMC7738049

